# Why Are MgC_3_H Isomers Missing in the Interstellar Medium?

**DOI:** 10.1021/acs.jpca.2c02220

**Published:** 2022-06-29

**Authors:** Sunanda Panda, Devipriya Sivadasan, Nisha Job, Aland Sinjari, Krishnan Thirumoorthy, Anakuthil Anoop, Venkatesan S. Thimmakondu

**Affiliations:** † Department of Chemistry, Indian Institute of Technology Kharagpur, Kharagpur 721 302, West Bengal, India; ‡ Department of Chemistry, School of Advanced Sciences, Vellore Institute of Technology, Vellore 632 014, Tamil Nadu, India; § School of Mathematics, Biological, Exercise & Physical Sciences, San Diego Miramar College, San Diego, California 92126-2910, United States; ∥ Department of Chemistry and Biochemistry, San Diego State University, San Diego, California 92182-1030, United States

## Abstract

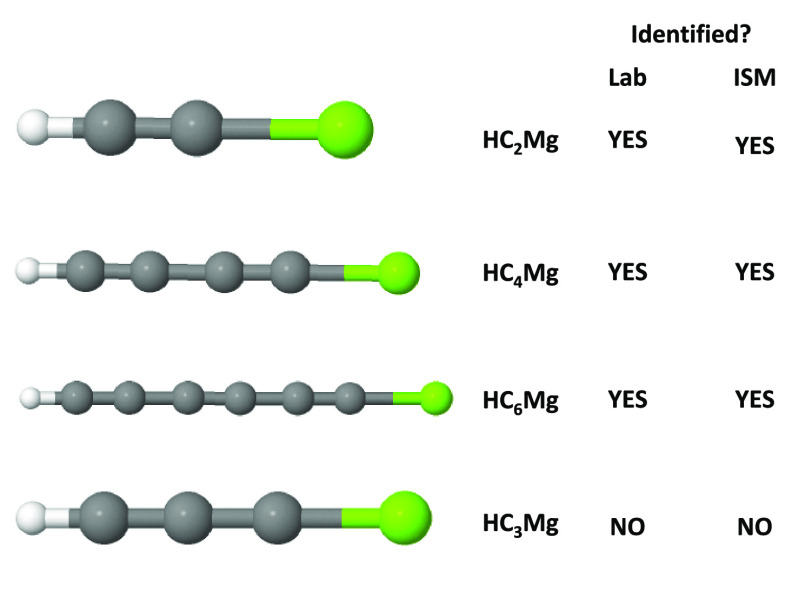

Considering the recent findings of linear doublet (^2^Σ^+^) MgC_*n*_H isomers (*n* = 2, 4, and 6) in the evolved carbon star IRC+10216, various structural isomers of MgC_3_H and MgC_3_H^+^ are theoretically investigated here. For MgC_3_H, 11 doublet and 8 quartet stationary points ranging from 0.0 to 71.8 and 0.0 to 110.1 kcal mol^–1^, respectively, have been identified initially at the UωB97XD/6-311++G(2d,2p) level. To get accurate relative energies, further energy evaluations are carried out for all isomers with coupled cluster methods and thermochemical modules such as G3//B3LYP, G4MP2, and CBS-QB3 methods. Unlike the even series, where the global minima are linear molecules with a Mg atom at one end, in the case of MgC_3_H, the global minimum geometry turns out to be a cyclic isomer, 2-magnesabicyclo[1.1.0]but-1,3,4-triyl (**1**, *C*_2*v*_, ^2^*A*_1_). In addition, five low-lying isomers, magnesium-substituted cyclopropenylidene (**2**, *C*_*s*_, ^2^*A*′), 1-magnesabut-2,3-dien-1-yl-4-ylidene (**3**, *C*_*s*_, ^2^*A*″), 1-magnesabut-2-yn-1-yl-4-ylidene (**4**, *C*_*s*_, ^2^*A*″), 2λ^3^-magnesabicyclo[1.1.0]but-1,3-diyl-4-ylidene (**5**, *C*_2*v*_;, ^2^*A*_1_), and 1-magnesabut-2,3-dien-2-yl-4-ylidene (**6**, *C*_*∞v*_, ^2^Σ^+^), were also identified. The doublet linear isomer of MgC_3_H, 1-magnesabutatrienyl (**10**, *C*_*∞v*_, ^2^Σ^+^) turns out to be a minimum but lies 54.1 kcal mol^–1^ above **1** at the ROCCSD(T)/cc-pVTZ level. The quartet (^4^Σ^+^) electronic state of **10** was also found to be a minimum, but it lies 8.0 kcal mol^–1^ above **1** at the same level. Among quartets, isomer **10** is the most stable molecule. The next quartet electronic state (of isomer **11**) is 34.4 kcal mol^–1^ above **10**, and all other quartet electronic states of other isomers are not energetically close to low-lying doublet isomers **2** to **6**. Overall, the chemical space of MgC_3_H contains more cyclic isomers (**1**, **2**, and **3**) on the low-energy side unlike their even-numbered MgC_*n*_H counterparts (*n* = 2, 4, and 6). Though the quartet electronic state of **10** is linear, it is not the global minimum geometry on the MgC_3_H potential energy surface. Isomerization pathways among the low-lying isomers (doublets of **1**–**4** and a quartet of **10**) reveal that these molecules are kinetically stable. For the cation, MgC_3_H^+^, the cyclic isomers (**1**^**+**^, **2**^**+**^, and **3**^**+**^) are on the low-energy side. The singlet linear isomer, **10**^**+**^, is a fourth-order saddle point. The low-lying cations are quite polar, with dipole moment values of >7.00 D. The current theoretical data would be helpful to both laboratory astrophysicists and radioastronomers for further studies on the MgC_3_H^0/+^ isomers.

## Introduction

Finding molecules many light years away is not only an open challenge to the scientific community but also an essential study that needs to be undertaken for a thorough understanding of star-forming regions, the formation of planets, and astrobiology.^1−9^ To confirm the molecules in an unambiguous manner in the interstellar medium (ISM) and circumstellar shells, laboratory rest frequencies are an essential prerequisite in most cases.^10−23^ Therefore, logically analyzing astrochemical issues requires a coordinated effort from experts across the scientific community, which includes radioastronomers, astrophysicists, molecular spectroscopists, organic chemists, electrical engineers, and quantum information scientists.^24−33^ As an example, let us take the case of a linear MgC_2_H radical. The pure rotational spectrum of 1-magnesaprop-2-yn-1-yl (MgC_2_H, ^2^Σ^+^) was recorded by Ziuyrs and co-workers in 1995.^34,35^ It was speculated at that time that MgC_2_H would be present in the ISM because it is isoelectronic with MgNC^36^ and MgCN.^37^ Through ab initio calculations, Woon suggested the formation pathways of MgC_2_H, MgC_2_H^+^, and cyclic-MgC_2_ in the ISM.^38,39^ Nearly two decades later, MgC_2_H was tentatively assigned in the evolved carbon star, IRC+10216, in 2014.^40^ Five years later, in 2019, the presence of MgC_2_H was finally confirmed along with the findings of MgC_4_H and MgC_3_N.^41^ It is noted here that the confirmation of MgNC (^2^Σ^+^) in IRC+10216 triggered interest in this molecule as well as in other organomagnesium compounds.^42−48^ MgNC has also been identified in two protoplanetary nebulae, CRL 2688 and CRL 618.^49,50^ The metastable isomer of the MgNC radical, MgCN, was also confirmed in IRC+10216 in 1995.^37^ In 2021, the presence of MgC_6_H (1-magnesahept-2,4,6-triyn-1-yl) was also confirmed.^51^ Naturally, these observations consequently provoke a question regarding the presence of odd series of MgC_*n*_H isomers (where, *n* = 3, 5, 7, etc.,) in IRC+10216 and/or in other interstellar sources. Thus, in this work, we have theoretically characterized the potential energy surfaces of MgC_3_H and MgC_3_H^+^ using density functional theory and coupled-cluster methods.

Though several experimental studies have been carried out to record the electronic transitions of MgC_2*n*_H (*n* = 1–3) in the visible region (in the gas phase),^52−55^ the number of experimental studies for the odd series MgC_2*n*–1_H (*n* ≥ 2) appears to be limited. In the past, Largo and co-workers carried out theoretical studies for MgC_3_ and MgC_3_H^+^ isomers at the MP2(full)/6-311G(d) and B3LYP/6-311G(d) levels.^56,57^ Dong et al. have observed a great number of magnesium carbon hydride clusters (Mg_*m*_C_*n*_H_*x*_) by the ablation of Mg foil into a mixture of 10% CH_4_/He expansion gas in their mass spectra.^58^ Notably, in their experiments, clusters with odd mass numbers (that is, systems containing an odd number of electrons) are detected, and no signal related to MgC_3_H isomers was observed.^58^ Graham and co-workers have recorded the vibrational spectrum of the linear MgC_3_^–^ anion using Fourier transform infrared (FTIR) spectroscopy.^59^

**Figure 1:**
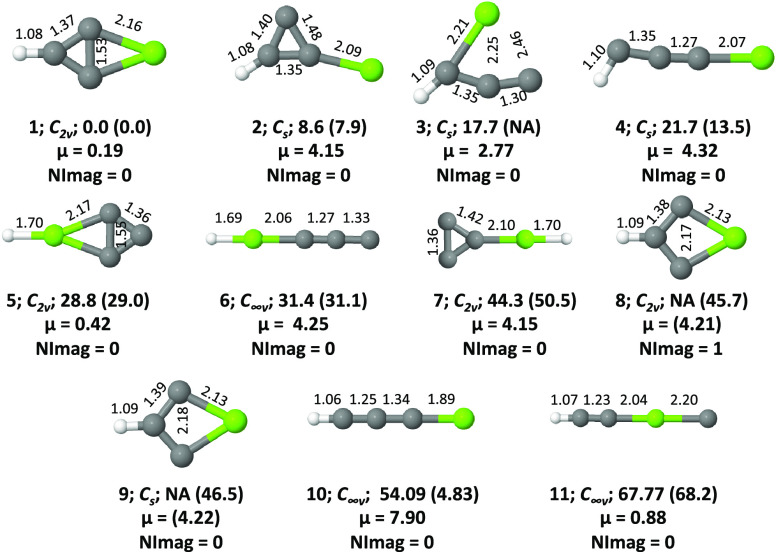
Isomers **1**–**11** of MgC_3_H in their doublet ground electronic states. Relative energies (ZPVE inclusive, in kcal mol^–1^) and dipole moments (in Debye) are calculated at the ROCCSD(T)/cc-pVTZ level. Values shown in parentheses are calculated at the CBS-QB3 level. The number of imaginary frequencies (NImag) obtained for each geometry is also given. Here, NA stands for not applicable, which means that the geometry either did not converge or led to some other geometry at that particular level.

**Figure 2:**
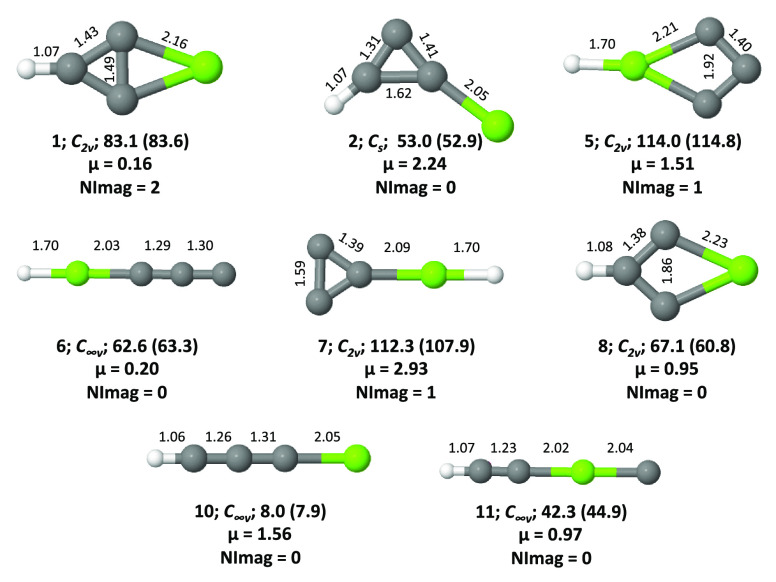
Isomers **1**–**11** of MgC_3_H in their quartet ground electronic states. Relative energies (ZPVE inclusive, in kcal mol^–1^) and dipole moments (in Debye) are calculated at the ROCCSD(T)/cc-pVTZ level. Values shown in parentheses are calculated at the CBS-QB3 level. The number of imaginary frequencies (NImag) obtained for each geometry is also given.

Theoretical studies including structures, energetics, and spectroscopic properties of MgC_3_H isomers have been missing until now in the literature. Thus, isomers of MgC_3_H in their doublet and quartet electronic states (Figures 1 and 2) are characterized for the first time in this work. Six low-lying doublet isomers, 2-magnesabicyclo[1.1.0]but-1,3,4-triyl (**1**), magnesium-substituted cyclopropenylidene (**2**), 1-magnesabut-2,3-dien-1-yl-4-ylidene (**3**), 1-magnesabut-2-yn-1-yl-4-ylidene (**4**), 2λ^3^-magnesabicyclo[1.1.0]but-1,3-diyl-4-ylidene (**5**), and 1-magnesabut-2,3-dien-2-yl-4-ylidene (**6**), and one low-lying quartet isomer, 1-magnesabutatrienyl (**10**), could be considered to be suitable target molecules for experimental observations. Unlike the MgC_*n*_H (where, *n* = 2, 4, 6, etc.) even series, where the global minima are linear molecules with the magnesium atom at one end, here for MgC_3_H, a cyclic isomer (**1**) was identified to be the global minimum geometry. Considering the presence of Mg^+^ and C_3_H (both prop-1-yn-3-ylidyne and cycloprop-1-yn-3-yl) radicals in the ISM,^60−65^ it is anticipated that isomers of MgC_3_H could also be present. However, the prerequisite for detecting these molecules many light years away is the availability of laboratory rest frequencies,^66−74^ but there appear to be no laboratory astrophysical studies for this system at the moment. Thus, computational studies are undertaken, which may aid not only the laboratory studies but also the radioastronomical observations.

## Computational Details

For the doublet and quartet electronic states of MgC_3_H and also for the triplet electronic states of MgC_3_H^+^, the geometry optimization calculations are initially carried out at the UωB97XD^75^/6-311++G(2d,2p)^76,77^ level. For the singlet electronic states of MgC_3_H^+^, calculations are made at the same level using restricted Hartree–Fock (RHF) wave functions. Vibrational frequencies (harmonic) are calculated for each stationary point to confirm whether it is a minimum, transition state, or *n*th-order saddle point. The number of imaginary frequencies (NImag) obtained for each stationary point is indicated underneath the geometry. To obtain better relative energies, single-point energy calculations are carried out using the coupled-cluster (CC) method at either the UCCSD(T)^78,79^/6-311++G(2d,2p) or RCCSD(T)/6-311++G(2d,2p)/ωB97XD^75^/6-311++G(2d,2p) level on top of the optimized geometries obtained from density functional theory (DFT). The ωB97XD functional has been purposefully chosen because it incorporates empirical dispersion corrections^80^ as well as long-range corrections. Full geometry optimization and frequency calculations for all MgC_3_H isomers (doublets and quartets) are also carried out at the ROCCSD(T)/cc-pVTZ level. To further evaluate the relative energies, calculations are made using the composite methods, G3//B3LYP,^81^ G4(MP2),^82,83^ and CBS-QB3.^84,85^ These relative energies are documented in Tables 1 and 2 for MgC_3_H and MgC_3_H^+^ isomers, respectively.

**Table 1:** ZPVE-Corrected Relative Energies of MgC_3_H Isomers in their Doublet and Quartet Ground Electronic States Calculated at Different Levels

	doublets										
	**1**	**2**	**3**	**4**	**5**	**6**	**7**	**8**	**9**	**10**	**11**
level	^2^*A*_1_	^2^*A*′	^2^*A*′	^2^*A*^″^	^2^*A*_1_	^2^Σ^+^	^2^*A*_1_	^2^*A*_1_	^2^*A*′	^2^Σ^+^	^2^Σ^+^
ROωB97XD/6-311++G(2d,2p)	0.0	6.9	28.0^d^	30.2	30.3	34.5	51.0	b	b	32.1	72.6
UωB97XD/6-311++G(2d,2p)	0.0	6.8	25.6^d^	28.1	29.1	32.3	49.6	52.5	52.8	54.1	71.8
UCCSD(T)/6-311++G(2d,2p)^a^	0.0	8.1	27.0^d^	14.3	31.9	33.1	55.3	49.4	49.4	54.2	65.2
G3//B3LYP	0.0	7.9	c	17.7	29.2	30.1	50.8	50.8	52.5	8.7^e^	66.2
G4MP2	0.0	7.8	c	18.5	29.2	33.2	51.1	51.4	52.1	59.8	68.1
ROCCSD(T)/cc-pVTZ	0.0	8.6	17.7	21.7	28.8	31.7	50.9	b	b	54.1	68.5
CBS-QB3	0.0	7.9	c	13.5	29.0	31.1	50.5	45.7	46.5	4.8^f^	68.2

	quartets										
	**1**	**2**	**3**	**4**	**5**	**6**	**7**	**8**	**9**	**10**	**11**
level	^4^*B*_1_	^4^*A*^″^			^4^*A*_2_	^4^Σ^+^	^4^*B*_1_	^4^*A*_2_		^4^Σ^+^	^4^Σ^+^
ROωB97XD/6-311++G(2d,2p)	87.5	54.0	c	c	120.7	69.3	118.7	63.5	g	13.1	47.5
UωB97XD/6-311++G(2d,2p)	85.0	51.4	c	c	116.2	61.0	107.9	58.8	g	6.1	46.4
UCCSD(T)/6-311++G(2d,2p)^a^	86.1	53.9	c	c	117.4	63.1	111.4	60.5	g	8.3	38.8
G3//B3LYP	83.0	52.3	c	c	113.5	62.2	107.3	60.7	g	6.6	41.5
G4MP2	84.3	54.3	c	c	116.4	67.0	110.0	63.0	g	10.9	45.6
ROCCSD(T)/cc-pVTZ	83.1	53.0	c	c	114.0	62.6	112.3	67.1	g	8.0	42.3
CBS-QB3	83.6	52.9	c	c	114.8	63.3	107.9	60.8	g	7.9	44.9

a Calculated at the UCCSD(T)/6-311++G(2d,2p)//UωB97XD/6-311++G(2d,2p) level.

b Geometry optimization at this level for isomer **8** or **9** leads to isomer **1**.

c Geometry optimization at this level for isomer **3** or **4** leads to isomer **10**.

d The electronic state is ^2^*A*″.

e The wave function is highly spin-contaminated (⟨*S*^2^⟩ = 1.799861) at this level, and thus the relative energies are not in order.

f The wave function is highly spin-contaminated (⟨*S*^2^⟩ = 1.796338) at this level, and thus the relative energies are not in order.

g Geometry optimization at this level for isomer **9** leads to isomer **8**.

**Table 2:** ZPVE-Corrected Relative Energies of MgC_3_H^+^ Isomers in their Singlet and Triplet Ground Electronic States Calculated at Different Levels

	singlets										
level	**1**	**2**	**3**	**4**	**5**	**6**	**7**	**8**	**9**	**10**	**11**
ωB97XD/6-311++G(2d,2p)	0.0	16.0	22.4	29.6	78.4	62.6	89.3	b	b	35.2	123.1
CCSD(T)/6-311++G(2d,2p)^a^	0.0	15.4	19.0	21.6	71.9	52.8	81.2	b	b	35.2	108.1
G3//B3LYP	0.0	14.7	19.3	19.0	70.7	51.6	80.6	b	b	33.7	110.6
G4MP2	0.0	14.2	19.7	18.3	69.1	52.4	80.5	b	b	35.2	111.0
CCSD(T)/cc-pVTZ	0.0	15.6	19.6	21.4	70.0	52.3	80.6	b	b	33.4	110.4
CBS-QB3	0.0	15.7	20.7	21.2	72.8	54.8	83.6	b	b	35.1	112.6

	triplets										
level	**1**	**2**	**3**	**4**	**5**	**6**	**7**	**8**	**9**	**10**	**11**
UωB97XD/6-311++G(2d,2p)	47.8	25.2	c	c	97.4	98.7	81.5	53.4		11.3	99.2
UCCSD(T)/6-311++G(2d,2p)^a^	47.2	23.3	c	c	96.6	95.6	80.8	54.7		13.7	87.9
G3//B3LYP	47.2	25.1	c	13.2	103.1	95.2	79.3	55.0		13.7	93.5
G4MP2	46.6	24.7	c	15.5	102.6	98.3	78.8	55.8		17.3	94.7
CBS-QB3	48.7	26.9	c	13.5	103.8	97.1	130.7	55.3		14.7	96.9

a Calculated at the (U)CCSD(T)/6-311++G(2d,2p)//U)ωB97XD/6-311++G(2d,2p) level.

b Geometry optimization at this level for isomer **8** or **9** leads to isomer **1**.

c Geometry optimization at this level for isomer **3** or **4** leads to isomer **10**.

For the low-lying doublet isomers, appropriate transition states have been identified at the UωB97XD/6-311++G(2d,2p) level, and isomerization pathways were confirmed through intrinsic reaction coordinate (IRC)^86,87^ calculations at the same level. To accurately determine the activation energy barriers, single-point energy calculations were made at the UCCSD(T)/6-311++G(2d,2p)//UωB97XD/6-311++G(2d,2p) level. To assess the multireference character, *T*_1_ diagnostic values^88^ have been calculated at the UCCSD/6-311++G(2d,2p)//UωB97XD/6-311++G(2d,2p) level of theory. To check the kinetic stability of isomers **1** (^2^*A*_1_) and **10** (^4^Σ^+^), *ab initio* molecular dynamics (AIMD) simulations using the atom-centered density matrix propagation (ADMP)^89^ method are carried out at the UωB97XD/6-311++G(2d,2p) level of theory. All DFT calculations, calculations that involve composite methods, and AIMD simulations are carried out with the Gaussian suite of programs.^90^ All coupled-cluster calculations have been carried out with the CFOUR (2.00 beta version) program package.^91^

## Results and Discussion

**Figure 3:**
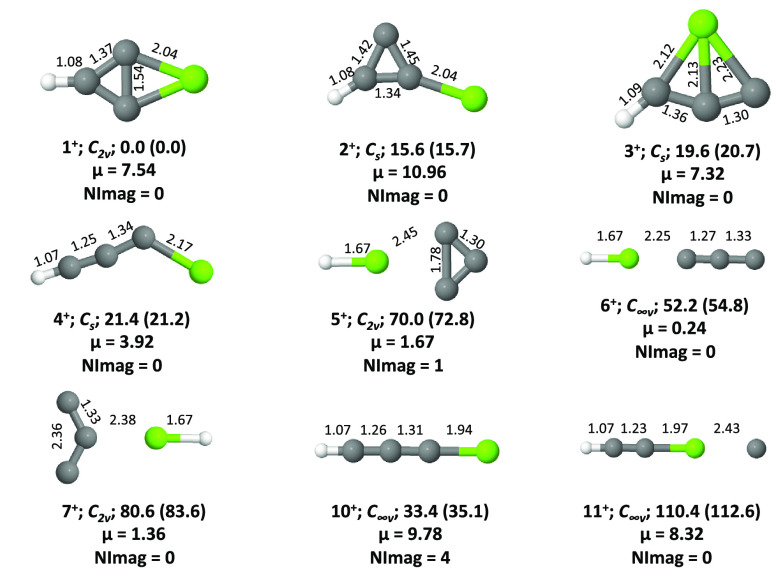
Isomers **1**–**11** of MgC_3_H^+^ in their singlet ground electronic states. ZPVE-corrected relative energies (in kcal mol^–1^) and dipole moments (in Debye) are calculated at the CCSD(T)/cc-pVTZ level. Values in parentheses are calculated at the CBS-QB3 level. The number of imaginary frequencies (NImag) obtained for each geometry is also given.

Optimized structures of isomers **1**–**11** of MgC_3_H^0/+^ in doublet, quartet, and singlet electronic states are shown in Figures 1, 2, and 3, respectively. For brevity, triplet electronic states of MgC_3_H^+^ are not shown here because all of them energetically lie above singlets. Zero-point vibrational energy (ZPVE)-corrected relative energies obtained at various levels for MgC_3_H and MgC_3_H^+^ isomers are shown in Tables 1 and 2, respectively. Various spectroscopic parameters of isomers **1**, **10**, and **2** are collected in Tables 3, 4, and 5, respectively.

**Table 3:** Inertial Axis Dipole Moment Components, Absolute Dipole Moment (in Debye), Rotational and Centrifugal Distortion Constants (in MHz), Harmonic Vibrational Frequencies (in cm^–1^), and IR Intensities (Given in Parentheses, in km mol^–1^) of the ^2^*A*_1_ Electronic State of Isomer **1** Calculated at Different Levels^a^

	6-311++G(2d,2p)		cc-pVTZ		
parameter	ROωB97XD	UωB97XD	ROCCSD(T)	UCCSD(T)	description
μ_a_	–0.1908	–0.1832	–0.1871	–0.1844	
μ_b_					
|μ_a_|	0.1908	0.1832	0.1871	0.1844	
*A*_e_	37 353.86	37 353.83	35 921.12	35 920.68	
*B*_e_	5040.82	5040.82	5023.66	5023.01	
*C*_e_	4441.45	4441.45	4407.29	4406.78	
Δ_*J*_			0.1886 × 10^–2^	0.1887 × 10^–2^	
Δ_*K*_			0.2392	0.2392	
Δ_*JK*_			0.2579 × 10^–1^	0.2584 × 10^–1^	
δ_*J*_			0.2545 × 10^–3^	0.2545 × 10^–3^	
δ_*K*_			0.1947 × 10^–1^	0.1950 × 10^–1^	
ω_1_ (*a*_1_)	3238.4 (1.6)	3238.7 (1.7)	3238.6 (1.6)	3238.6 (1.6)	C–H stretch
ω_2_ (*a*_1_)	1620.3 (18.7)	1620.3 (18.7)	1577.1 (15.7)	1577.1 (15.7)	C–C–C stretch
ω_3_ (*a*_1_)	876.9 (35.2)	876.8 (35.4)	825.9 (29.1)	825.8 (29.1)	C–C stretch
ω_4_ (*a*_1_)	451.9 (106.1)	451.9 (106.0)	463.9 (94.2)	463.8 (94.2)	Mg–C_2_ stretch
ω_5_ (*b*_1_)	886.6 (1.2)	887.0 (1.2)	862.5 (1.3)	862.4 (1.3)	C–H wagging (out of plane)
ω_6_ (*b*_1_)	232.4 (8.1)	232.9 (8.0)	225.9 (6.5)	225.9 (6.5)	C–C–C twist (out of plane)
ω_7_ (*b*_2_)	1346.8 (5.6)	1347.0 (5.6)	1329.9 (4.6)	1329.9 (4.6)	CCC bend (in plane)
ω_8_ (*b*_2_)	1007.2 (9.9)	1007.4 (9.9)	1010.7 (10.7)	1010.7 (10.7)	C–H wagging (in plane)
ω_9_ (*b*_2_)	271.5 (60.3)	271.6 (60.2)	287.6 (52.2)	287.4 (52.2)	CCMg bend (in plane)

a Centrifugal distortion constants are from the *A*-reduced Hamiltonian.

**Table 4:** Inertial Axis Dipole Moment Components, Absolute Dipole Moment (in Debye), Rotational and Centrifugal Distortion Constants (in MHz), Harmonic Vibrational Frequencies (in cm^–1^), and IR Intensities (in Parentheses, in km mol^–1^) of the ^4^Σ^+^ Electronic State of Isomer **10** Calculated at Different Levels

	6-311++G(2d,2p)		cc-pVTZ		
parameter	ROωB97XD	UωB97XD	ROCCSD(T)	UCCSD(T)	description
μ_a_	1.7020	1.6977	1.5565	1.5837	
μ_b_					
|μ_a_|	1.7020	1.6977	1.5565	1.5837	
*B*_e_	2396.12	2390.48	2359.18	2359.85	
*D*_*J*_			0.2704 × 10^–3^	0.2694 × 10^–3^	
*D*_*K*_			0.2704 × 10^–3^	0.2694 × 10^–3^	
*D*_*JK*_			–0.5408 × 10^–3^	–0.5387 × 10^–3^	
ω_1_ (σ_g_^+^)	3460.9 (78.1)	3451.8 (70.8)	3439.8 (74.5)	3445.4 (76.2)	C–H stretch
ω_2_ (σ_g_^+^)	1664.5 (0.5)	1661.8 (4.9)	1618.5 (2.1)	1661.1 (4.7)	C–C stretch
ω_3_ (σ_g_^+^)	1270.8 (41.0)	1322.3 (40.6)	1283.5 (36.2)	1288.3 (36.2)	C–C–Mg stretch
ω_4_ (σ_g_^+^)	439.4 (99.0)	439.0 (97.3)	438.3 (84.4)	439.2 (83.8)	C–Mg stretch
ω_5_ (π)	455.6 (5.7)	449.5 (0.3)	556.8 (39.5)	444.7 (0.7)	C–C–H bend
ω_6_ (π)	439.2 (49.5)	292.9 (52.1)	507.9 (4.9)	183.0 (46.1)	C–C–H bend
ω_7_ (π)	119.1 (11.5)	118.5 (10.9)	123.3 (20.1)	114.3 (7.6)	C–C–Mg bend

**Table 5:** Inertial Axis Dipole Moment Components, Absolute Dipole Moment (in Debye), Rotational and Centrifugal Distortion Constants (in MHz), Harmonic Vibrational Frequencies (in cm^–1^), and IR Intensities (in Parentheses, in km mol^–1^) of ^2^*A*′ Electronic State of Isomer **2** Calculated at Different Levels^a^

	6-311++G(2d,2p)		cc-pVTZ		
parameter	ROωB97XD	UωB97XD	ROCCSD(T)	UCCSD(T)	description
μ_a_	–3.9832	3.9723	3.1792	3.1531	
μ_b_	–1.9716	–1.9572	2.6699	2.6659	
|μ_a_|	4.4444	4.4284	4.1516	4.1291	
*A*_e_	36 269.11	36 269.12	35 529.29	35 543.24	
*B*_e_	3730.78	3730.78	3718.44	3722.60	
*C*_e_	3382.81	3382.81	3366.15	3369.68	
*D*_*J*_			0.5380 × 10^–2^	0.7064 × 10^–2^	
*D*_*K*_			–0.5034	–0.9187	
*D*_*JK*_			0.6839	1.1186	
*D*_1_			–0.5432 × 10^–3^	–0.7347 × 10^–3^	
*D*_2_			–0.4687 × 10^–3^	–0.7684 × 10^–3^	
ω_1_ (*a*′)	3231.7 (4.07)	3231.7 (4.0)	3227.0 (4.5)	3227.4 (4.5)	C–H stretch
ω_2_ (*a*′)	1640.7 (9.8)	1639.9 (8.2)	1606.5 (13.6)	1607.3 (13.5)	C=C stretch
ω_3_ (*a*′)	1302.3 (22.2)	1301.6 (23.7)	1281.1 (15.0)	1281.2 (15.0)	C–C–H bend
ω_4_ (*a*′)	976.7 (10.0)	975.5 (10.5)	947.6 (9.8)	947.8 (9.7)	CCC bend
ω_5_ (*a*′)	940.8 (19.1)	941.1 (18.7)	925.4 (17.6)	925.7 (17.8)	C–H wag (in plane)
ω_6_ (*a*′)	411.3 (50.5)	410.4 (48.6)	426.8 (46.5)	427.4 (46.5)	C–Mg bend
ω_7_ (*a*′)	87.9 (15.8)	88.4 (15.5)	45.6 (15.2)	38.9 (15.3)	C_3_H kink
ω_8_ (*a*″)	891.7 (6.0)	892.0 (6.1)	868.0 (6.2)	867.8 (6.2)	C–H wag (out of plane)
ω_9_ (*a*″)	216.2 (0.1)	216.1 (0.0)	208.7 (0.1)	207.7 (0.2)	CCMg bend (out of plane)

a Centrifugal distortion constants are from the *S*-reduced Hamiltonian.

### Energetics

#### MgC_3_H

After analyzing the doublet and quartet electronic states of various isomers of MgC_3_H, one could arrive at a conclusion that the doublet electronic state (^2^*A*_1_) of 2-magnesabicyclo[1.1.0]but-1,3,4-triyl (**1**) is the most stable isomer at various levels (see Table 1). From the energetic perspective, the first six isomers of the doublet (**1**–**6**) and the quartet of isomer **10** lie within 1.5 eV (∼34.6 kcal mol^–1^). Therefore, the possibility of finding these molecules in the laboratory is quite high. Among the doublets, isomers **1** (μ = 0.19 D) and **5** (μ = 0.42 D) are less polar whereas **2**, **3**, **4**, and **6** are quite polar, with dipole moment values of 4.15, 2.77, 4.32, and 4.25 D, respectively. Isomer **10** of the quartet is also moderately polar (μ = 1.56 D). Though the doublet of **10** is highly polar (μ = 7.90 D), it lies 54.1 kcal mol^–1^ above **1** at the ROCCSD(T)/cc-pVTZ level. It is noted here that the relative energies obtained with respect to G3//B3LYP and CBS-QB3 methods for the doublet electronic state of isomer **10** are not in good agreement compared with other methods (see Table 1). The reason for this discrepancy is due to a large amount of spin contamination in both the G3//B3LYP and CBS-QB3 methods. Both methods use the UB3LYP functional for the geometry optimization. Ideally, for a doublet electronic state, the ⟨*S*^2^⟩ value should be 0.75. However, the values we obtained for isomer **10** were 1.799861 and 1.796338, respectively. The initial wave function is certainly very badly spin-contaminated; consequently, the energies are not in the correct order compared to other levels. We did do a stability analysis of the optimized geometries of **10** obtained through the G3//B3LYP and CBS-QB3 methods.^92^ It is noted here that the wave functions are symmetry-broken and show UHF instabilities.^93^ For these reasons, the energies reported with respect to the G3//B3LYP and CBS-QB3 methods for isomer **10** are unreliable. Thus, we rely on coupled-cluster data and other levels of theory for the relative energy of this isomer.

From the thermodynamic stability point of view, the quartet of isomer **10** is the second-most-stable isomer because it lies 8.0 kcal mol^–1^ above the doublet of **1**. The doublet of isomer **2** is the third-most-stable isomer because it lies at 8.6 kcal mol^–1^ above **1**. Therefore, further emphasis is mostly given to these three molecules (doublets of **1** and **2** and a quartet of **10**) instead of all low-lying isomers. It is noted here that, among the doublets, geometry **8** is a transition state and all others are minima at the ROCCSD(T)/cc-pVTZ level. In the case of quartets, geometry optimization of isomers **3** and **4** leads to isomer **10**, whereas isomer **9** geometry optimization leads to isomer **8**. Therefore, in total, merely eight stationary points were found within the quartet electronic states.

On the basis of the multireference characteristics of C_3_H isomers discussed elsewhere,^65^ we suspected that some of the isomers of MgC_3_H may have multireference characteristics. To clarify this, we have computed the *T*_1_ diagnostic values^88^ for all isomers at the UCCSD/6-311++G(2d,2p)//UωB97XD/6-311++G(2d,2p) level. Calculated *T*_1_ diagnostic values for isomers **1**, **2**, **5**, and **11** were 0.013, 0.014, 0.019, and 0.017, respectively. Because these values are less than 0.020, one can state that these isomers are not multireference in character. For isomer **7**, the value obtained was 0.022, which is slightly above the threshold. For isomers **3**, **4**, **6**, **8**, **9**, and **10**, the calculated *T*_1_ values were 0.118, 0.162, 0.052, 0.139, 0.141, and 0.058, respectively. The quartet of isomer **10** also shows moderate multireference character with a *T*_1_ value of 0.039. Although we have not carried out multireference coupled-cluster (MRCC) or complete active space self-consistent field calculations on any of these isomers, we note that such a study is deferred to future work.

#### MgC_3_H^+^

The relative stabilities of both singlet and triplet electronic states of MgC_3_H^+^ isomers (**1**^**+**^–**11**^**+**^) have been calculated at different levels, and they are collected in Table 2. Like the doublet neutral radicals, on the cation PES, the singlet electronic state of 2-magnesabicyclo[1.1.0]but-1,3,4-triyl (**1**^**+**^) is the most stable isomer. The singlets of isomers **2**^**+**^, **3**^**+**^, and **4**^**+**^ are 15.6, 19.6, and 21.4 kcal mol^–1^ above the singlet of **1**^**+**^, respectively, at the CCSD(T)/cc-pVTZ level. The singlet linear isomer, **10**^**+**^, is a fourth-order saddle point, and isomer **5**^**+**^ turned out to be a transition state at the latter level. Geometry optimization of **8**^**+**^ and **9**^**+**^ at all levels led to **1**^**+**^. All of the stationary points on the cation PES had higher polarity due to the positive charge. The dipole moment values of the first four isomers (**1**^**+**^–**4**^**+**^) are 7.84, 12.32, 7.32, and 7.93 D, respectively. Therefore, apart from neutrals, the cations are also suitable candidates for microwave spectroscopic and radioastronomical studies. It is noted here that the triplet electronic states of all cations are above the singlet electronic state of **1**^**+**^.

### 2-Magnesabicyclo[1.1.0]but-1,3,4-triyl (**1**)

**Figure 4:**
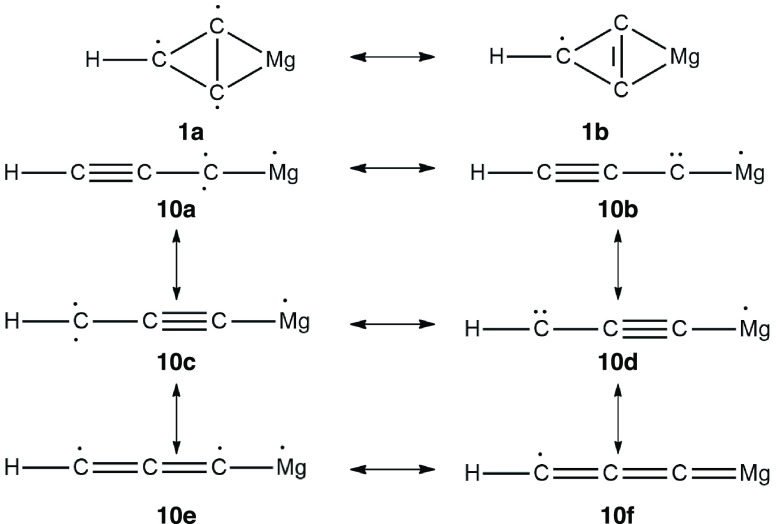
Valence structures of isomers **1** and **10** of MgC_3_H.

The point-group symmetry of **1** is *C*_2*v*_, and the ground electronic state is ^2^*A*_1_. The quartet electronic state (^4^*B*_1_) of **1** is a second-order saddle point and is 83.1 kcal mol^–1^ above the doublet of **1**. Though the former is energetically the most stable molecule for MgC_3_H at all levels studied here, it remains elusive in the laboratory to date. The binding energy (BE) for **1** (BE = *E*_MgC_3_H_ – (*E*_c–C_3_H_ + *E*_Mg_)) is −47.2 kcal mol^–1^ at the (U)ωB97XD/6311++G(2d,2p) level of theory. Therefore, the molecule is sufficiently bound and the Mg–C bonds are not extremely weak. The transannular C–C bond length obtained at all levels (1.53 to 1.54 Å) reveals that it exhibits single-bond characteristics. Therefore, resonance structure **1a** is more dominant than **1b** (see Figure 4). Though isomer **1** is less polar (μ = 0.19 D), in principle, it can be identified through rotational spectroscopy. We note that there are weakly polar van der Waals complexes such as the Ne–Ar dimer whose dipole moment is just 0.0022 D, yet pure rotational transitions have been measured.^94^ Equilibrium rotational constants *A*_e_, *B*_e_, and *C*_e_ obtained from the optimized geometries at different levels (see Table 3) reveal that this molecule is an asymmetric top. In addition, one can also detect isomer **1** through IR spectroscopy. Although two of the low-frequency modes ν_9_ (287.6 cm^–1^, CCMg bending) and ν_4_ (463.9 cm^–1^, Mg–C_2_ stretching) are very intense, one cannot confirm these modes easily. On the other hand, ν_8_ (1010.7 cm^–1^, C–H wagging) and ν_2_ (1577.0 cm^–1^, CCC stretching) modes could readily be seen (see Table 3).

### 1-Magnesabutatrienyl (**10**)

The ground electronic state of **10** is ^4^Σ^+^. It lies 8.0 kcal mol^–1^ above **1** of the doublet at the ROCCSD(T)/cc-pVTZ level. This *C*_*∞v*_-symmetric linear molecule is the second-most-stable isomer for MgC_3_H, and it is ∼8 times polar than **1** with a dipole moment value of 1.56 D. However, this molecule also remains elusive in the laboratory to date. The binding energy (BE) for **10** (BE = *E*_MgC_3_H_ – (*E*_l–C_3_H_ + *E*_Mg_)) is −45.4 kcal mol^–1^ at the (U)ωB97XD/6-311++G(2d,2p) level of theory. This implies that the Mg atom is sufficiently bound to the carbon atom. The BE values we have calculated for isomers **1** and **10** indirectly support the relative energy ordering we have obtained for these two isomers because the most stable isomer has the highest binding energy. Six different valence structures are shown in Figure 4 for isomer **10**. Three structures (**10a**, **10c**, and **10e**) represent the quartet electronic state and the other three (**10b**, **10d**, and **10f**) represent the doublet electronic state. On the basis of the bond lengths obtained, it is evident that valence structure **10a** is dominant because the C–C bond length of 1.26 Å (connected to the H atom) exhibits triple-bond characteristics whereas the other C–C bond length is intermediate between a double and a triple bond. The rotational and centrifugal distortion constants obtained at various levels are shown in Table 4. The C–H stretching (ν_1_) and C–C–Mg stretching (ν_3_) modes whose frequencies are 3439.8 and 1283.5 cm^–1^, respectively, at the ROCCSD(T)/cc-pVTZ level could readily be seen in the IR spectra.

### Magnesium-Substituted Cyclopropenylidene (**2**)

The point-group symmetry of **2** is *C*_*s*_, and the ground electronic state is ^2^*A*′. This molecule is 8.6 kcal mol^–1^ above the doublet of **1** and is the third-most-stable isomer on the MgC_3_H potential energy surface. The quartet electronic state (^4^*A*″) of **2** is also a minimum, but it lies 53.0 kcal mol^–1^ above **1**. Among the three low-lying minima, isomer **2** is the highest polar molecule with a dipole moment value of 4.15 D at the ROCCSD(T)/cc-pVTZ level. Therefore, the chances of identifying this molecule both in the laboratory and in the ISM are high. Moreover, the inertial axis dipole moment components are in two directions for this molecule (see Table 5), and thus both *a*- and *b*-type rotational transitions are possible. Moreover, from equilibrium rotational constants *A*_e_, *B*_e_, and *C*_e_, one could infer that this molecule is closely approaching the prolate limit because the difference between the *B*_e_ and *C*_e_ rotational constants is not very high. The C=C stretching (ν_2_) and CCH bending (ν_3_) modes whose frequencies are 1606.5 and 1281.1 cm^–1^, respectively, at the ROCCSD(T)/cc-pVTZ level could readily be seen in the IR spectra.

### Other Low-Lying Isomers of MgC_3_H^0/+^

Apart from the doublet electronic states of isomers **1** and **2** and the quartet electronic state of isomer **10**, the doublet electronic states of isomers **3**, **4**, and **5** of MgC_3_H could be considered to be low-lying isomers based on the relative energies (below 30 kcal mol^–1^). Except **5**, which is less polar, the other three isomers are quite polar. Likewise, for the cation, apart from singlet **1**^**+**^, the three other singlet isomers (**2**^**+**^–**4**^**+**^) could be considered to be low-lying isomers. Therefore, the chances of identifying these molecules both in the laboratory and in the ISM are moderate.

### Isomerization Pathways

**Figure 5:**
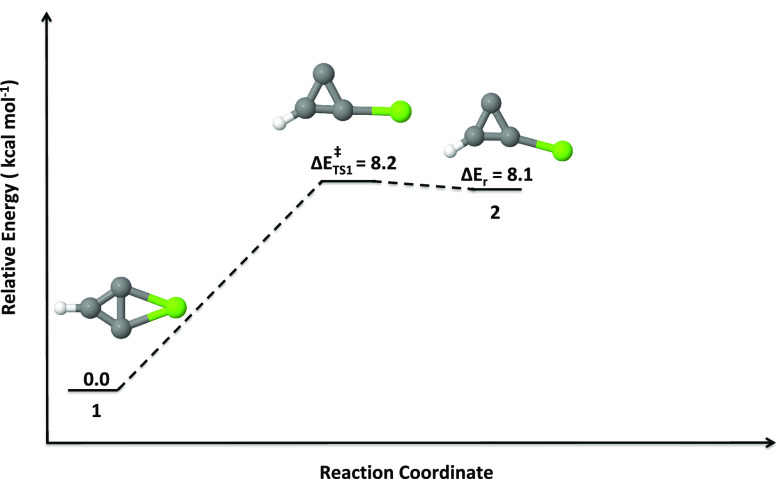
Isomerization pathway of isomer **1** to **2** (doublets). Relative energies are calculated at the UCCSD(T)/6-311++G(2d,2p)//UωB97XD/6-311++G(2d,2p) level.

**Figure 6:**
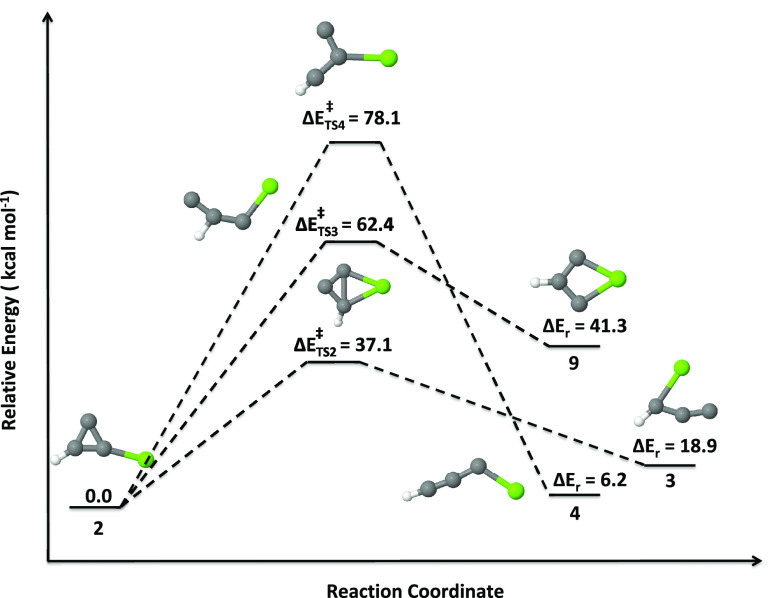
Isomerization pathway of isomer **2** to others (**3**, **4**, and **9**; doublets). Relative energies are calculated at the UCCSD(T)/6-311++G(2d,2p)//UωB97XD/6-311++G(2d,2p) level.

**Figure 7:**
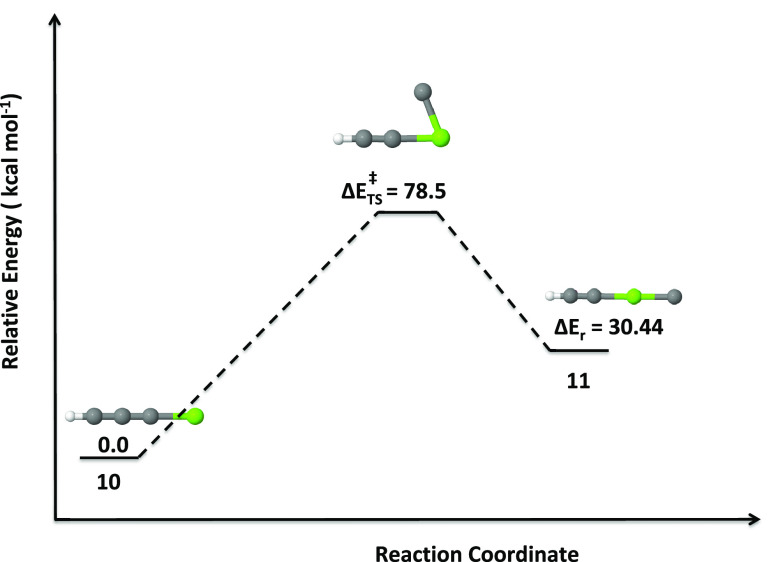
Isomerization pathway of isomer **10** to **11** (quartets). Relative energies are calculated at the UCCSD(T)/6-311++G(2d,2p)//UωB97XD/6-311++G(2d,2p) level.

To assess the kinetic stability of the low-lying doublet (**1**–**4**) and quartet (**10** and **11**) isomers, transition states have been identified initially at the UωB97XD/6-311++G(2d,2p) level, and the minimum-energy pathways are confirmed through IRC calculations at the same level. To accurately determine the activation energy barriers, single-point energy calculations are carried out at the UCCSD(T)/6-311++G(2d,2p)//UωB97XD/6-311++G(2d,2p) level. In Figures 5–7, a schematic outline of the isomerization pathways of isomers **1** to **2**, **2** to **3** (as well as **4** and **9**), and **10** to **11**, respectively, is shown. Altogether, five different transition states (**TS1**–**TS5**) have been identified. The dissociation of the C–Mg bond (either one) of **1** leads to **TS1** with an activation energy of 8.2 kcal mol^–1^. The reaction energy calculated for the isomerization of **1** to **2** is just 8.1 kcal mol^–1^. This indirectly implies that the reverse process (**2** to **1**) is nearly barrierless. Therefore, isomer **1** is certainly a kinetically stable molecule.

For the isomerization of **2**, three different transition states (**TS2**, **TS3**, and **TS4**; see Figure 6) have been identified. By bringing the C–Mg bond closer to the carbene carbon of **2**, one could get a puckered four-membered transition state with a trans-annular C–C bond. The activation energy for **TS2** is 37.1 kcal mol^–1^. IRC calculations from this lead to isomer **3** with a reaction energy of 18.9 kcal mol^–1^. We also identified two more transition states by breaking the C–C single bonds of the cyclopropenylidene ring of **2**. The activation energies for **TS3** and **TS4** are 62.4 and 78.1 kcal mol^–1^, respectively. These are high-energy transition states; therefore, the conversions of **2** to **9** and **2** to **4** are unlikely to occur. The isomerization of **2** to **3** is also unlikely because it requires an activation of energy of 37.1 kcal mol^–1^. However, isomerization of **2** to **1** is barrierless and thus **2** is not a kinetically stable molecule. Next, we turned our attention to the low-lying quartet isomers (**10** and **11**) because the quartet of isomer **10** is the second-most-stable molecule thermodynamically. It is also worth mentioning here that among the quartets, isomer **10** is the most stable molecule. Bending of the C–Mg bond of **10** leads to **TS5** and thus the isomerization of **10** to **11** requires an activation energy of 78.5 kcal mol^–1^, which is quite high. Therefore, we conclude that the quartet linear isomer of **10** is not only a thermodynamically stable but also a kinetically stable molecule.

### *Ab Initio* Molecular Dynamics

**Figure 8:**
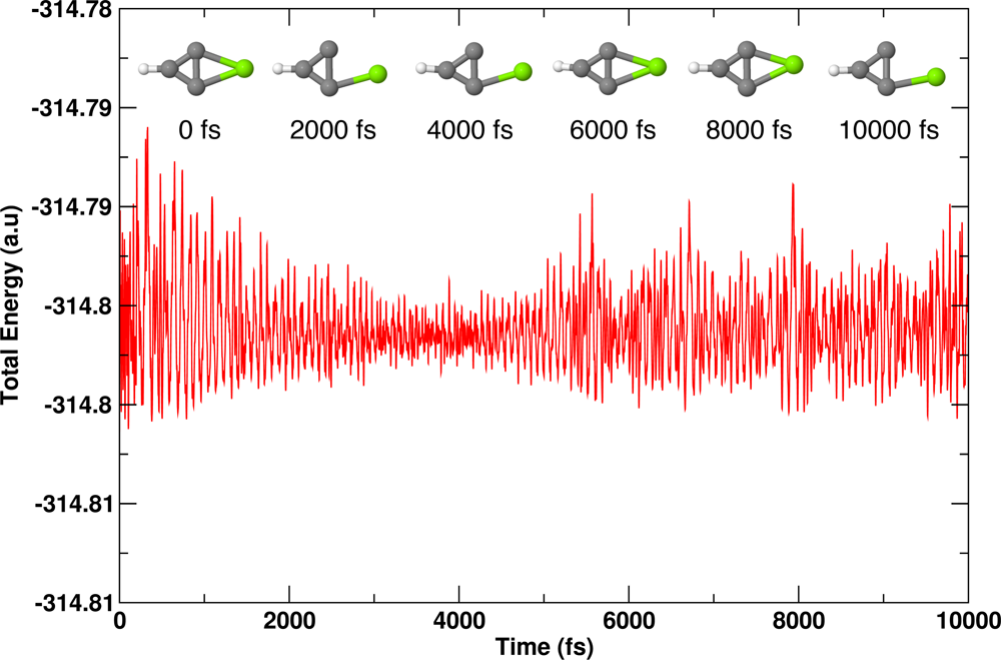
Energy evolution of isomer **1** (^2^*A*_1_) of MgC_3_H obtained from the AIMD simulation carried out at 298 K and 1 atm pressure for 10 000 fs at the UωB97XD/6-311++G(2d,2p) level.

**Figure 9:**
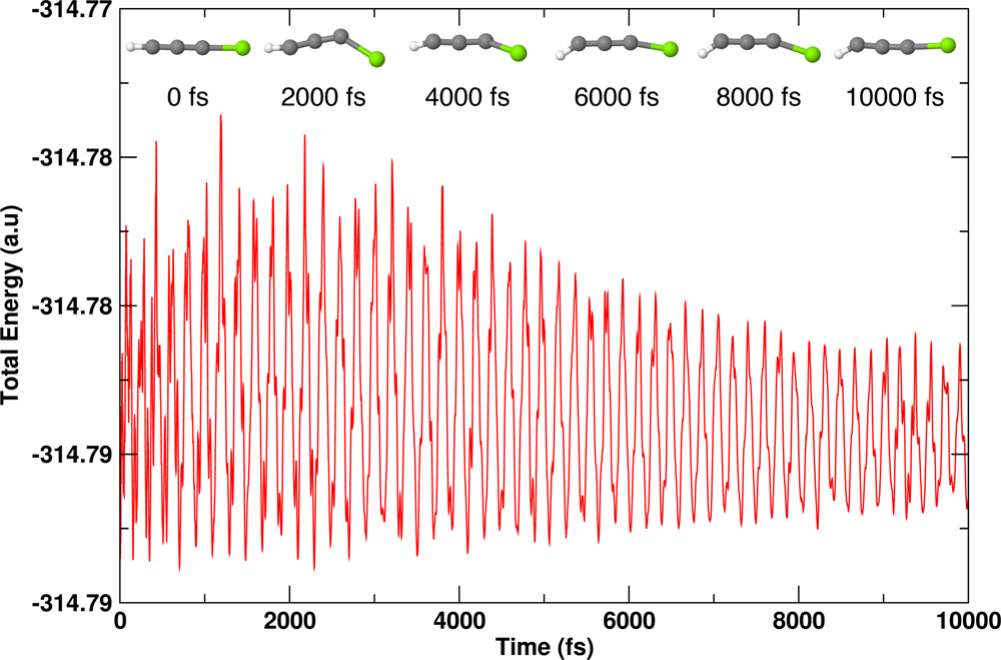
Energy evolution of isomer **10** (^4^Σ^+^) of MgC_3_H obtained from the AIMD simulation carried out at 298 K and 1 atm pressure for 10 000 fs at the UωB97XD/6-311++G(2d,2p) level.

Apart from identifying transition-state geometries and confirming the minimum-energy paths through IRC calculations, we have also carried out *abinitio* molecular dynamics simulations to reaffirm the kinetic stability of two of the lowest lying isomers of MgC_3_H (a doublet of **1** and a quartet of **10**). These calculations were made using the ADMP^89^ approach as incorporated in the Gaussian 16 program.^90^ These simulations were carried out at 298 K and 1 atm pressure for 10 000 fs. For isomers **1** and **10**, the time evolutions of total energies are shown in Figures 8 and 9, respectively. To show the geometric changes that are happening to each isomer over 10 000 fs, snapshots at 2000 fs interval are added. These figures show a balanced oscillation in the energies and also firmness in the geometries over the entire time period. Therefore, one can further conclude that these molecules are indeed kinetically stable.

## Conclusions

Various isomers of MgC_3_H^0/+^ are studied in this work, which are systems of potential interstellar interest, by using DFT, coupled-cluster, and thermochemical modules. For both MgC_3_H^0/+^, the most stable isomer turns out to be isomer **1** (**1**^**+**^), which is a cyclic molecule. This is a stark contrast compared to the even-numbered MgC_*n*_H isomers (*n* = 2, 4, etc.), where the most stable isomers are linear molecules. Linear isomer **10** within the quartet electronic state (^4^Σ^+^) was found to be the second-most-stable isomer thermodynamically at various levels. Though the doublet of **10** turns out to be a minimum at the ROCCSD(T)/cc-pVTZ level, it lies 54.1 kcal mol^–1^ above the doublet electronic state of **1**. The doublet of **10** is certainly the most polar molecule with a dipole moment of 7.90 D. However, it lies in the high-energy region. Considering the presence of cyclic-C_3_H, linear-C_3_H, and Mg^+^ in the ISM, it is postulated here that the formation of isomers **1** and **10** is plausible. Though isomer **1** is less polar (μ = 0.19 D), the detection of **1** in the laboratory should be possible. On the other hand, the quartet electronic state of isomer **10** is moderately polar (μ = 1.55 D). Therefore, identifying isomer **10** in the laboratory may be more straightforward than identifying isomer **1**. Among the three low-lying minima, isomer **2** is highly polar with a dipole moment value of 4.15 D. However, **2** is not kinetically stable at all because the isomerization pathway of **1** to **2** indicates that it will quickly revert back to **1**. Therefore, the detection of this molecule in the laboratory will be very challenging. It is also noted here that the doublet electronic states of isomers **3**, **4**, and **6** are quite polar with dipole moments of 7.45, 3.04, and 4.38 D, respectively. Therefore, the detection of these molecules is also possible though they are on the high-energy side. Isomerization pathways indicate that the three low-lying isomers (doublets of **1** and **4** and a quartet of **10**) are kinetically stable molecules. For MgC_3_H^+^, the cyclic isomers (**1**^**+**^, **2**^**+**^, and **3**^**+**^) are on the low-energy side. The singlet linear isomer, **10**^**+**^, turned out to be a fourth-order saddle point at the CCSD(T)/cc-pVTZ level. Moreover, the low-lying cations are quite polar with dipole moment values of >7.00 D. Therefore, they are also suitable candidates for laboratory detection by rotational spectroscopy. To conclude, the structural, energetic, and rotational spectroscopic parameters computed in this work would aid both molecular spectroscopists and radioastronomers in the future.

## Supporting Information Available

Cartesian coordinates of the optimized geometries, total electronic energies, and zero-point vibrational energies calculated at different levels; optimal geometry parameters obtained from DFT, harmonic vibrational frequencies, infrared intensities, and anharmonic vibrational frequencies for two of the low-lying isomers (PDF)
