# Supplementary material for: Why Are MgC3H Isomers Missing in the Interstellar Medium?
Source: J Phys Chem A. 2022 Jun 29;126(27):4465–75. doi: 10.1021/acs.jpca.2c02220 (PMC9382639; doi:10.1021/acs.jpca.2c02220)
Supplement: Supplementary file 1 — jp2c02220_si_001.pdf [file jp2c02220_si_001.pdf]

Supporting information for: Why Are MgC_3H Isomers Missing in the Interstellar Medium?

Sunanda Panda,[†] Devipriya Sivadasan,[‡] Nisha Job,[‡] Aland Sinjari,[¶] Krishnan Thirumoorthy,[‡] Anakuthil Anoop,[†] and Venkatesan S. Thimmakondu[*,§]

†*Department of Chemistry, Indian Institute of Technology Kharagpur, Kharagpur 721 302, West Bengal, India*

‡*Department of Chemistry, School of Advanced Sciences, Vellore Institute of Technology, Vellore - 632 014, Tamil Nadu, India*

¶*School of Mathematics, Biological, Exercise & Physical Sciences, San Diego Miramar College, San Diego, CA, 92126-2910, USA*

§*Department of Chemistry and Biochemistry, San Diego State University, San Diego, CA 92182-1030, USA*

E-mail: vthimmakondusamy@sdsu.edu

Contents

List of Figures

S1 Isomers **1-11** of MgC_3H in their respective doublet ground electronic states. ZPVE-corrected relative energies (in kcal mol^{-1}) and dipole moments (in Debye) are calculated at the UωB97XD/6-311++G(2d,2p) level of theory. . . . S4

S2 Isomers **1-11** of MgC_3H in their respective quartet ground electronic states. ZPVE-corrected relative energies (in kcal mol^{-1}) and dipole moments (in Debye) are calculated at the UωB97XD/6-311++G(2d,2p) level of theory. . . . S6

S3 Energy evolution of isomer **3** of MgC_3H obtained from the AIMD simulation carried out at 298 K and 1 atm pressure for 10000 fs at the UωB97XD/6-311++G(2d,2p) level. S15

S4 Energy evolution of isomer **4** of MgC_3H obtained from the AIMD simulation carried out at 298 K and 1 atm pressure for 10000 fs at the UωB97XD/6-311++G(2d,2p) level. S16

List of Tables

S1 Total energies (E), Zero-point vibrational energies ($ZPVE$), $ZPVE$-corrected total energies (E+$ZPVE$), absolute dipole moments ($|\mu|$), relative energies (ΔE), ZPVE-corrected relative energies (ΔE+$ZPVE$), $< S^2 >$, and number of imaginary frequencies (NImag) of isomers **1** to **11** of MgC_3H in their respective doublet ground electronic states calculated at the UωB97XD/6-311++G(2d,2p) level of theory. S5

S2 Computed energies and properties of MgC_3H isomers in their quartet ground electronic states calculated at the UωB97XD/6-311++G(2d,2p) level of theory. S7

S3 Computed energies (in kcal mol^{-1}) of various of isomers of MgC_3H in their respective doublet ground electronic states calculated at the G3//B3LYP level. S8

S4 Computed energies (in kcal mol^{-1}) of various of isomers of MgC_3H in their respective doublet ground electronic states calculated at the G4MP2 level. . S9

S5 Computed energies (in kcal mol^{-1}) of various of isomers of MgC_3H in their respective doublet ground electronic states calculated at the CBS-QB3 level. S10

S6 Computed energies (in kcal mol^{-1}) of various of isomers of MgC_3H in their respective quartet ground electronic states calculated at the G3//B3LYP level. S11

S7 Computed energies (in kcal mol^{-1}) of various of isomers of MgC_3H in their respective quartet ground electronic states calculated at the G4MP2 level. . S12

S8 Computed energies (in kcal mol^{-1}) of various of isomers of MgC_3H in their respective quartet ground electronic states calculated at the CBS-QB3 level. S13

S9 Total energies (E), Zero-point vibrational energies ($ZPVE$), $ZPVE$-corrected total energies (E+$ZPVE$), absolute dipole moments ($|\mu|$), relative energies (ΔE), ZPVE-corrected relative energies (ΔE+$ZPVE$), and number of imaginary frequencies (NImag) of isomers **1** to **11** of MgC_3H^+ in their respective singlet ground electronic states calculated at the CCSD(T)/cc-pVTZ level of theory. S14

S10 Rotational Constants (in MHz), Inertial Axis Dipole Moment Components, and Absolute Dipole Moments (in Debye), and Centrifugal Distortion Constants of MgC_3H Isomers (Doublets) Calculated at the fc-ROCCSD(T)/cc-pVTZ Level of Theory S17

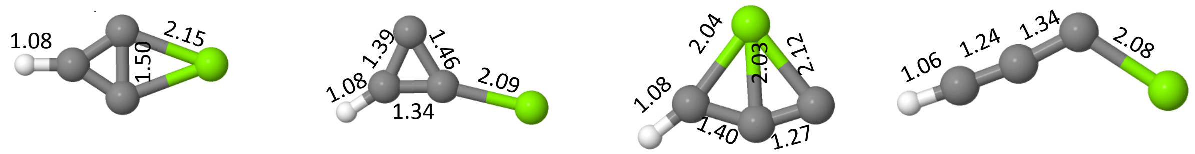

1; C_{2v}; 0.00
μ = 0.18
NImag = 0

2; C_s; 6.84
μ = 4.43
NImag = 0

3; C_s; 25.59
μ = 7.45
NImag = 0

4; C_s; 28.10
μ = 3.04
NImag = 0

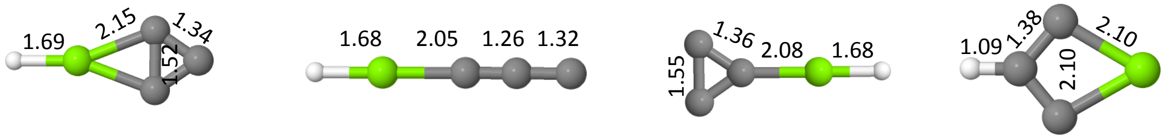

5; C_{2v}; 29.11
μ = 0.45
NImag = 0

6; $C_{\infty v}$; 32.31
μ = 4.38
NImag = 0

7; C_{2v}; 49.55
μ = 4.32
NImag = 1

8; C_{2v}; 52.53
μ = 5.49
NImag = 1

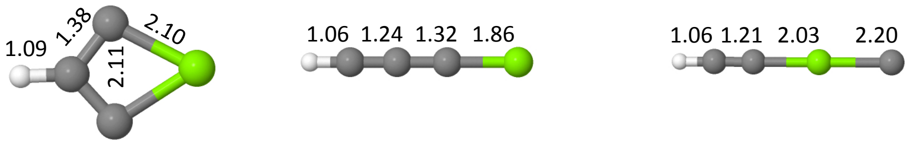

9; C_s; 52.75
μ = 5.48
NImag = 0

10; $C_{\infty v}$; 54.10
μ = 9.62
NImag = 1

11; $C_{\infty v}$; 71.84
μ = 1.19
NImag = 0

Figure S1: Isomers **1-11** of MgC_3H in their respective doublet ground electronic states. ZPVE-corrected relative energies (in kcal mol^{-1}) and dipole moments (in Debye) are calculated at the UωB97XD/6-311++G(2d,2p) level of theory.

Table S1: Total energies (E), Zero-point vibrational energies ($ZPVE$), $ZPVE$-corrected total energies (E+$ZPVE$), absolute dipole moments ($|\mu|$), relative energies (ΔE), ZPVE-corrected relative energies (ΔE+$ZPVE$), $< S^2 >$, and number of imaginary frequencies (NImag) of isomers **1** to **11** of MgC_3H in their respective doublet ground electronic states calculated at the UωB97XD/6-311++G(2d,2p) level of theory.

Isomer	Point group	E a.u	$ZPVE$ a.u	E+$ZPVE$ a.u	$\|\mu\|$ Debye	ΔE kcal mol^{-1}	ΔE+$ZPVE$ kcal mol^{-1}	$< S^2 >$	NImag
**1**	C_{2v}	-314.802267	0.022630	-314.779636	0.18	0.00	0.00	0.750560	0
**2**	C_s	-314.790834	0.022091	-314.768743	4.43	7.17	6.84	0.751622	0
**3**	C_s	-314.760452	0.021603	-314.738849	7.45	26.24	25.59	0.804742	0
**4**	C_s	-314.754976	0.020120	-314.734857	3.04	29.68	28.10	0.785647	0
**5**	C_{2v}	-314.749379	0.016125	-314.733254	0.45	33.19	29.11	0.757367	0
**6**	$C_{\infty v}$	-314.743691	0.015549	-314.728142	4.38	36.76	32.31	0.772174	0
**7**	C_{2v}	-314.712826	0.012147	-314.700679	4.32	56.12	49.55	0.759290	1
**8**	C_{2v}	-314.715400	0.019478	-314.695922	5.49	54.51	52.53	0.843943	1
**9**	C_s	-314.715479	0.019908	-314.695571	5.48	54.46	52.75	0.847996	0
**10**	$C_{\infty v}$	-314.714212	0.020790	-314.693422	9.62	55.26	54.10	0.821099	1
**11**	$C_{\infty v}$	-314.684650	0.019502	-314.665149	1.19	73.81	71.84	0.755248	0

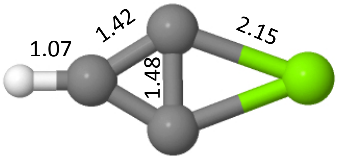

1; C_{2v}; 78.86
μ = 0.06
NImag = 2

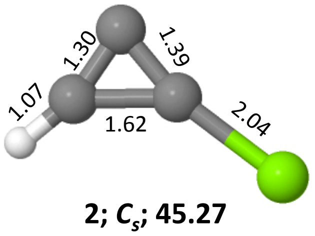

2; C_s; 45.27
μ = 2.57
NImag = 0

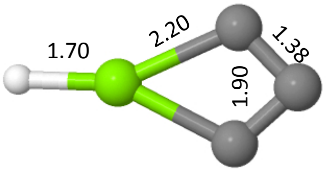

5; C_{2v}; 110.11
μ = 0.60
NImag = 1

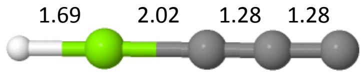

6; $C_{\infty v}$; 54.89
μ = 0.71
NImag = 0

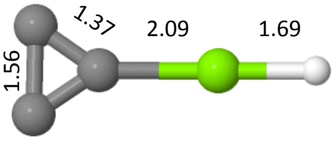

7; C_{2v}; 101.80
μ = 3.41
NImag = 1

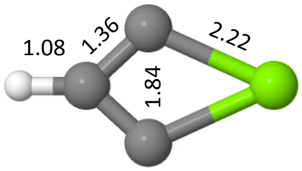

8; C_{2v}; 52.66
μ = 0.80
NImag = 0

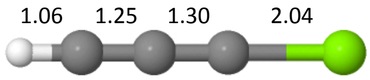

10; $C_{\infty v}$; 0.00
μ = 1.70
NImag = 0

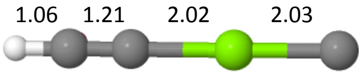

11; $C_{\infty v}$; 40.25
μ = 1.01
NImag = 0

Figure S2: Isomers **1-11** of MgC_3H in their respective quartet ground electronic states. ZPVE-corrected relative energies (in kcal mol^{-1}) and dipole moments (in Debye) are calculated at the UωB97XD/6-311++G(2d,2p) level of theory.

Table S2: Computed energies and properties of MgC_3H isomers in their quartet ground electronic states calculated at the UωB97XD/6-311++G(2d,2p) level of theory.

Isomer	Point group	E a.u	$ZPVE$ a.u	$E+ZPVE$ a.u	$\|\mu\|$ Debye	ΔE kcal mol^{-1}	$\Delta E+ZPVE$ kcal mol^{-1}	$< S^2 >$	NImag
**10**	$C_{\infty v}$	-314.7894660	0.019585	-314.769882	1.70	0.00	0.00	3.821183	0
**11**	$C_{\infty v}$	-314.7256432	0.019907	-314.705736	1.01	40.05	40.25	3.755624	0
**2**	C_s	-314.7169885	0.019246	-314.697742	2.57	45.48	45.27	3.767088	0
**8**	C_{2v}	-314.7050148	0.019050	-314.685965	0.80	52.99	52.66	3.805557	0
**6**	$C_{\infty v}$	-314.6979435	0.015530	-314.682413	0.71	57.43	54.89	3.841692	0
**1**	C_{2v}	-314.6615465	0.017331	-314.644216	0.06	80.27	78.86	3.764492	2
**7**	C_{2v}	-314.6196588	0.012003	-314.607656	3.41	106.56	101.80	3.811041	1
**5**	C_{2v}	-314.6084784	0.014074	-314.594404	0.60	113.57	110.11	3.797622	1

Table S3: Computed energies (in kcal mol^{-1}) of various of isomers of MgC_3H in their respective doublet ground electronic states calculated at the G3//B3LYP level.

Isomer	E (0 K)	E (298.15 K)	H (298.15 K)	G (298.15 K)
**1**	-314.592979	-314.588676	-314.587732	-314.619418
**2**	-314.580327	-314.575669	-314.574725	-314.608499
**3**	–	–	–	–
**4**	-314.564792	-314.560090	-314.559145	-314.592664
**5**	-314.546513	-314.541587	-314.540643	-314.573317
**6**	-314.545096	-314.539495	-314.538551	-314.571695
**7**	-314.511961	-314.506632	-314.505688	-314.539693
**8**	-314.512020	-314.508056	-314.507112	-314.538275
**9**	-314.509310	-314.504779	-314.503835	-314.536717
**10**	-314.579095	-314.574230	-314.573286	-314.606245
**11**	-314.487434	-314.481588	-314.480643	-314.514855
Isomer	ΔE (0 K)	ΔE (298.15 K)	ΔH (298.15 K)	ΔG (298.15 K)
**1**	0.00	0.00	0.00	0.00
**2**	7.94	8.16	8.16	6.85
**3**	–	–	–	–
**4**	17.69	17.94	17.94	16.79
**5**	29.16	29.55	29.55	28.93
**6**	30.05	30.86	30.86	29.95
**7**	50.84	51.48	51.48	50.03
**8**	50.80	50.59	50.59	50.92
**9**	52.50	52.65	52.65	51.90
**10**	8.71	9.07	9.07	8.27
**11**	66.23	67.20	67.20	65.61

Table S4: Computed energies (in kcal mol^{-1}) of various of isomers of MgC_3H in their respective doublet ground electronic states calculated at the G4MP2 level.

Isomer	E (0 K)	E (298.15 K)	H (298.15 K)	G (298.15 K)
**1**	-314.3726160	-314.3683870	-314.3674430	-314.3989850
**2**	-314.3601410	-314.3555520	-314.3546080	-314.3881740
**3**	-	-	-	-
**4**	-314.3432000	-314.3378420	-314.3368970	-314.3718340
**5**	-314.3261620	-314.3213000	-314.3203560	-314.3529010
**6**	-314.3197360	-314.3141790	-314.3132350	-314.3463070
**7**	-314.2911950	-314.2859260	-314.2849820	-314.3188490
**8**	-314.2907610	-314.2868830	-314.2859380	-314.3169380
**9**	-314.2896110	-314.2851210	-314.2841770	-314.3170210
**10**	-314.2772870	-314.2729170	-314.2719720	-314.3023900
**11**	-314.2641310	-314.2583800	-314.2574360	-314.2914640
Isomer	ΔE (0 K)	ΔE (298.15 K)	ΔH (298.15 K)	ΔG (298.15 K)
**1**	0.00	0.00	0.00	0.00
**2**	7.83	8.05	8.05	6.78
**3**	-	-	-	-
**4**	18.46	19.17	19.17	17.04
**5**	29.15	29.55	29.55	28.92
**6**	33.18	34.02	34.02	33.06
**7**	51.09	51.75	51.75	50.29
**8**	51.36	51.14	51.15	51.49
**9**	52.09	52.25	52.25	51.43
**10**	59.82	59.91	59.91	60.61
**11**	68.08	69.03	69.03	67.47

Table S5: Computed energies (in kcal mol^{-1}) of various of isomers of MgC_3H in their respective doublet ground electronic states calculated at the CBS-QB3 level.

Isomer	E (0 K)	E (298.15 K)	H (298.15 K)	G (298.15 K)
**1**	-314.197013	-314.192758	-314.191814	-314.223409
**2**	-314.184401	-314.179816	-314.178872	-314.212377
**3**	–	–	–	–
**4**	-314.175546	-314.170499	-314.169555	-314.203585
**5**	-314.150882	-314.146022	-314.145077	-314.177616
**6**	-314.147486	-314.141840	-314.140896	-314.174206
**7**	-314.116581	-314.111324	-314.110380	-314.144191
**8**	-314.124169	-314.120292	-314.119348	-314.150346
**9**	-314.122848	-314.118331	-314.117387	-314.150324
**10**	-314.189319	-314.183237	-314.182293	-314.217856
**11**	-314.088342	-314.082538	-314.081594	-314.115773
Isomer	ΔE (0 K)	ΔE (298.15 K)	ΔH (298.15 K)	ΔG (298.15 K)
**1**	0.00	0.00	0.00	0.00
**2**	7.91	8.12	8.12	6.92
**3**	-	-	-	-
**4**	13.47	13.97	13.97	12.44
**5**	28.95	29.33	29.33	28.74
**6**	31.08	31.95	31.95	30.88
**7**	50.47	51.10	51.10	49.71
**8**	45.71	45.47	45.47	45.85
**9**	46.54	46.70	46.70	45.86
**10**	4.83	5.97	5.97	3.48
**11**	68.19	69.16	69.16	67.54

Table S6: Computed energies (in kcal mol^{-1}) of various of isomers of MgC_3H in their respective quartet ground electronic states calculated at the G3//B3LYP level.

Isomer	E (0 K)	E (298.15 K)	H (298.15 K)	G (298.15 K)
**10**	-314.582540	-314.578009	-314.577064	-314.608959
**11**	-314.526853	-314.521211	-314.520267	-314.554445
**2**	-314.509635	-314.504570	-314.503626	-314.538374
**8**	-314.496189	-314.492027	-314.491083	-314.523335
**6**	-314.493918	-314.488535	-314.487591	-314.520926
**1**	-314.460684	-314.456536	-314.455592	-314.487668
**7**	-314.421965	-314.416467	-314.415523	-314.450798
**5**	-314.412035	-314.407773	-314.406828	-314.439182
Isomer	ΔE (0 K)	ΔE (298.15 K)	ΔH (298.15 K)	ΔG (298.15 K)
**10**	0.00	0.00	0.00	0.00
**11**	34.94	35.64	35.64	34.21
**2**	45.75	46.08	46.08	44.29
**8**	54.19	53.95	53.95	53.73
**6**	55.61	56.15	56.15	55.24
**1**	76.47	76.23	76.22	76.11
**7**	100.76	101.37	101.37	99.25
**5**	106.99	106.82	106.82	106.54

Table S7: Computed energies (in kcal mol^{-1}) of various of isomers of MgC_3H in their respective quartet ground electronic states calculated at the G4MP2 level.

Isomer	E (0 K)	E (298.15 K)	H (298.15 K)	G (298.15 K)
**10**	-314.355280	-314.350733	-314.349789	-314.381695
**11**	-314.299954	-314.294392	-314.293448	-314.327478
**2**	-314.286090	-314.281150	-314.280206	-314.314679
**8**	-314.272241	-314.268135	-314.267191	-314.299321
**6**	-314.265781	-314.260420	-314.259476	-314.292780
**1**	-314.238231	-314.234139	-314.233195	-314.265158
**7**	-314.197318	-314.191856	-314.190912	-314.226121
**5**	-314.187137	-314.182916	-314.181972	-314.214239
Isomer	ΔE (0 K)	ΔE (298.15 K)	ΔH (298.15 K)	ΔG (298.15 K)
**10**	0.00	0.00	0.00	0.00
**11**	34.72	35.35	35.35	34.02
**2**	43.42	43.66	43.66	42.05
**8**	52.11	51.83	51.83	51.69
**6**	56.16	56.67	56.67	55.80
**1**	73.45	73.16	73.16	73.13
**7**	99.12	99.70	99.70	97.62
**5**	105.51	105.31	105.31	105.08

Table S8: Computed energies (in kcal mol^{-1}) of various of isomers of MgC_3H in their respective quartet ground electronic states calculated at the CBS-QB3 level.

Isomer	E (0 K)	E (298.15 K)	H (298.15 K)	G (298.15 K)
**10**	-314.184458	-314.178893	-314.177949	-314.211615
**11**	-314.125426	-314.119821	-314.118877	-314.153024
**2**	-314.112771	-314.107686	-314.106742	-314.141545
**8**	-314.100182	-314.096050	-314.095106	-314.127296
**6**	-314.096187	-314.090789	-314.089845	-314.123232
**1**	-314.063816	-314.059706	-314.058762	-314.090760
**7**	-314.025041	-314.019629	-314.018685	-314.053680
**5**	-314.014033	-314.009812	-314.008868	-314.041137
Isomer	ΔE (0 K)	ΔE (298.15 K)	ΔH (298.15 K)	ΔG (298.15 K)
**10**	0.00	0.00	0.00	0.00
**11**	37.04	37.07	37.07	36.77
**2**	44.98	44.68	44.68	43.97
**8**	52.88	51.98	51.98	52.91
**6**	55.39	55.29	55.29	55.46
**1**	75.70	74.79	74.79	75.84
**7**	100.04	99.94	99.94	99.11
**5**	106.94	106.10	106.10	106.98

Table S9: Total energies (E), Zero-point vibrational energies ($ZPVE$), $ZPVE$-corrected total energies (E+$ZPVE$), absolute dipole moments ($|\mu|$), relative energies (ΔE), ZPVE-corrected relative energies ($\Delta E+ZPVE$), and number of imaginary frequencies (NImag) of isomers **1** to **11** of MgC_3H^+ in their respective singlet ground electronic states calculated at the CCSD(T)/cc-pVTZ level of theory.

Isomer	Point group	E a.u	$ZPVE$ kcal mol^{-1}	$E+ZPVE$ a.u	$\|\mu\|$ Debye	ΔE kcal mol^{-1}	$\Delta E+ZPVE$ kcal mol^{-1}	NImag
**1**	C_{2v}	-313.93284872	14.4756	-313.90978040	7.54	0.0	0.0	0
**2**	C_s	-313.90696672	13.7917	-313.88498827	10.96	16.2	15.6	0
**3**	C_s	-313.90001121	13.4822	-313.87852598	7.32	20.6	19.6	0
**4**	C_s	-313.89586797	12.6334	-313.87573538	3.92	23.2	21.4	0
**5**	C_{2v}	-313.81244770	8.8942	-313.79827390	1.67	75.5	70.0	1
**6**	$C_{\infty v}$	-313.84137154	9.2883	-313.82656971	0.24	57.4	52.2	0
**7**	C_{2v}	-313.79455099	8.3250	-313.78128427	1.36	86.8	80.6	0
**10**	$C_{\infty v}$	-313.87428889	11.1707	-313.85648726	9.78	36.8	33.4	4
**11**	$C_{\infty v}$	-313.75262461	11.7429	-313.73391112	8.32	113.1	110.4	0

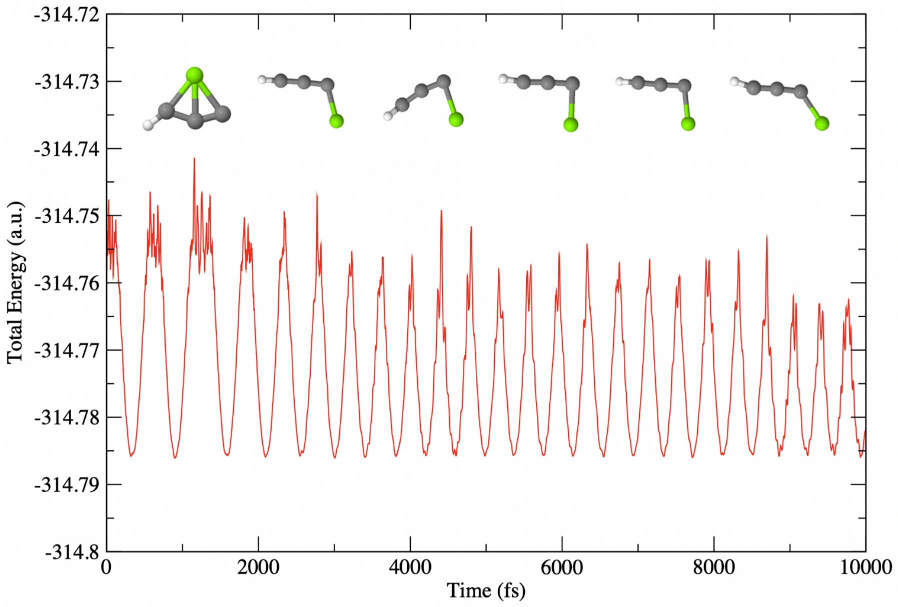

Figure S3: Energy evolution of isomer **3** of MgC_3H obtained from the AIMD simulation carried out at 298 K and 1 atm pressure for 10000 fs at the UωB97XD/6-311++G(2d,2p) level.

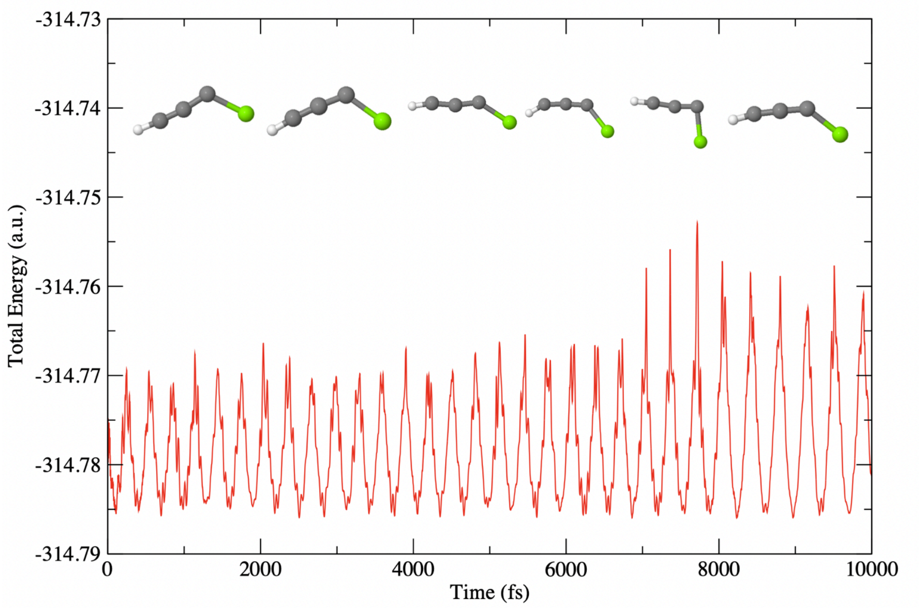

Figure S4: Energy evolution of isomer **4** of MgC_3H obtained from the AIMD simulation carried out at 298 K and 1 atm pressure for 10000 fs at the UωB97XD/6-311++G(2d,2p) level.

Table S10: Rotational Constants (in MHz), Inertial Axis Dipole Moment Components, and Absolute Dipole Moments (in Debye), and Centrifugal Distortion Constants of MgC_3H Isomers (Doublets) Calculated at the fc-ROCCSD(T)/cc-pVTZ Level of Theory

isomer	A_e	B_e	C_e	μ_a	μ_b	$\|\mu\|$	Δ_J	Δ_K	Δ_{JK}	δ_J	δ_K
**1**	35921.12	5023.66	4407.29	-0.19	–	0.19	0.1886×10^{-2}	0.2397	0.2509×10^{-1}	0.2544×10^{-3}	0.1912×10^{-1}
**2**[a]	35529.29	3718.44	3366.15	3.18	2.67	4.15	0.5380×10^{-2}	-0.5034	0.6839	-0.5432×10^{-3}	-0.4687×10^{-3}
**3**	11529.91	7958.18	4708.37	1.37	2.41	2.77	0.1178×10^{-1}	-0.2885×10^{-1}	0.2661×10^{-1}	0.4735×10^{-2}	0.3122×10^{-1}
**4**	530231.06	2371.93	2361.37	4.28	-0.61	4.32					
**5**	35223.95	4934.46	4328.14	-0.42	–	0.42	0.1837×10^{-2}	0.2263	0.2890×10^{-1}	0.2456×10^{-3}	0.2051×10^{-1}
**6**	–	2337.28	–	4.25	–	4.25	0.2601×10^{-3}	0.2601×10^{-3}	-0.5202×10^{-3}	–	–
**7**[b]											
**10**	–	2517.83	–	7.90	–	7.90	0.2288×10^{-3}	0.2288×10^{-3}	-0.4577×10^{-3}	–	–
**11**	–	2229.72	–	0.88	–	0.88	0.1491×10^{-3}	0.1491×10^{-3}	-0.2981×10^{-3}	–	–

[a] Centrifugal distortion constants are from S-reduced Hamiltonian as they are approaching nearly close to the prolate limit. [b] Transition state.

Cartesian coordinates (in Å units) of the optimized geometries of isomers **1** to **11** MgC_3H in their respective doublet ground electronic states obtained at the Uωb97xd/6-311++G(2D,2P) level of theory.

```
    5
isomer 1 (p1a) scf done:  -314.802267
 C     0.021328    -0.000000     1.000000
 H     1.099633    -0.000000     1.000000
 C    -1.104615    -0.000000     1.750819
 C    -1.104615     0.000000     0.249181
Mg    -3.122206     0.000000     1.000000

    5
isomer 2 (c1a) scf done:  -314.790834
 H    -0.156104     0.000000     0.027289
 C    -0.005418     0.000000     1.095262
 C     0.872466     0.000000     2.105926
 C    -0.560332     0.000000     2.365030
Mg     2.575721    -0.000000     3.315565

    5
isomer 3 (c5a) scf done:  -314.760452
 C     0.080590     0.000000    -0.170663
 C     0.312908     0.000000     1.206714
Mg     2.083571    -0.000000     0.213662
 C     1.183156    -0.000000     2.127485
 H    -0.894577     0.000000    -0.632823

    5
isomer 4 (c1e) scf done:  -314.754977
Mg    -0.442460     0.000000    -0.063483
 C     0.220301     0.000000     1.909992
 C     1.536653    -0.000000     2.156854
 C     2.715194    -0.000000     2.533703
 H     3.719857    -0.000000     2.877868

    5
isomer 5 (p1b) scf done:  -314.749379
Mg     0.105212    -0.000000     1.000000
 H     1.793860    -0.000000     1.000000
 C    -1.908924     0.000000     1.761477
 C    -1.908924    -0.000000     0.238523
 C    -3.013902    -0.000000     1.000000

    8
isomer 6 (c4d) scf done:  -314.743691
 C     0.092870     0.000000     1.000000
 C     1.413895     0.000000     1.000000
 C     2.672322     0.000000     1.000000
Mg     4.722846     0.000000     1.000000
 H     6.407796     0.000000     1.000000
XX     1.413895     1.000000     1.000000
XX     2.672322     1.000000     1.000000
XX     4.722846     1.000000     1.000000
```

```
    6
isomer 7 (c2a) scf done:  -314.712826
Mg     0.001987    -0.000000     1.000000
 H     1.686644    -0.000000     1.000000
 C    -2.081538     0.000000     1.000000
 C    -3.206371     0.000000     1.772679
 C    -3.206371    -0.000000     0.227321
XX     0.001987    -0.000000     2.000000

    5
isomer 8 (c5b) scf done:  -314.715400
 H    -0.000000     0.000000    -0.127162
 C    -0.000000     0.000000     0.958138
Mg     0.000000    -0.000000     3.666480
 C    -1.049218     0.000000     1.851387
 C     1.049218    -0.000000     1.851387

    5
isomer 9 (c5c) scf done:  -314.715479
 H    -0.197206     0.000000    -0.118606
 C    -0.215880     0.000000     0.966987
Mg    -0.109242     0.000000     3.651924
 C    -0.315623     1.053157     1.850845
 C    -0.315623    -1.053157     1.850845

    8
isomer 10 (c4a) scf done:  -314.714212
Mg     0.332043     0.000000     1.000000
 C     2.192599     0.000000     1.000000
 C     3.516562     0.000000     1.000000
 C     4.754116     0.000000     1.000000
 H     5.814409     0.000000     1.000000
XX     3.516562     1.000000     1.000000
XX     2.192599     1.000000     1.000000
XX     4.754116     1.000000     1.000000

    8
isomer 11 (c4b) scf done:  -314.684650
 C    -0.149423     0.000000     1.000000
Mg     2.047556     0.000000     1.000000
 C     4.074740     0.000000     1.000000
 C     5.286013     0.000000     1.000000
 H     6.350844     0.000000     1.000000
XX     2.047556     1.000000     1.000000
XX     4.074740     1.000000     1.000000
XX     5.286013     1.000000     1.000000
```

Cartesian coordinates (in Å units) of the optimized geometries of isomers **1** to **11** MgC_3H in their respective quartet ground electronic states obtained at the Uωb97xd/6-311++G(2D,2P) level of theory.

```
    8
isomer 10 (c4a) scf done:  -314.789467
Mg     0.196994     0.000000     1.000000
 C     2.235550     0.000000     1.000000
 C     3.536092     0.000000     1.000000
 C     4.789644     0.000000     1.000000
 H     5.851450     0.000000     1.000000
XX     3.536092     1.000000     1.000000
XX     2.235550     1.000000     1.000000
XX     4.789644     1.000000     1.000000

    8
isomer 11 (c4b) scf done:  -314.725643
 C    -0.010008     0.000000     1.000000
Mg     2.021613     0.000000     1.000000
 C     4.037688     0.000000     1.000000
 C     5.248068     0.000000     1.000000
 H     6.312367     0.000000     1.000000
XX     2.021613     1.000000     1.000000
XX     4.037688     1.000000     1.000000
XX     5.248068     1.000000     1.000000

    5
isomer 2 (c1a) scf done:  -314.716989
 H     0.015771     0.000000     0.002378
 C    -0.104237     0.000000     1.064495
 C     0.758591     0.000000     2.435043
 C    -0.617070     0.000000     2.261864
Mg     2.673278    -0.000000     3.145293

    5
isomer 8 (c5b) scf done:  -314.705015
 H    -0.000000     0.000000    -0.232905
 C    -0.000000     0.000000     0.848847
Mg     0.000000    -0.000000     3.873705
 C    -0.921772     0.000000     1.855292
 C     0.921772    -0.000000     1.855292

    8
isomer 6 (c4d) scf done:  -314.697944
 C     0.122172     0.000000     1.000000
 C     1.406832     0.000000     1.000000
 C     2.682275     0.000000     1.000000
Mg     4.703160     0.000000     1.000000
 H     6.395290     0.000000     1.000000
XX     1.406832     1.000000     1.000000
XX     2.682275     1.000000     1.000000
XX     4.703160     1.000000     1.000000

    5
isomer 1 (p1a) scf done:  -314.661547
```

```
 C     0.075432    -0.000000     1.000000
 H     1.143982    -0.000000     1.000000
 C    -1.137520    -0.000000     1.739558
 C    -1.137520     0.000000     0.260442
Mg    -3.154850     0.000000     1.000000

    5
isomer 7 (c2a) scf done:  -314.619659
Mg     0.000000     0.000000     1.595459
 H     0.000000     0.000000     3.281338
 C    -0.000000     0.000000    -0.491899
 C    -0.000000     0.777833    -1.622954
 C    -0.000000    -0.777833    -1.622954

    5
isomer 5 (p1b) scf done:  -314.608478
Mg    -0.000000     0.000000     1.291051
 H    -0.000000     0.000000     2.986161
 C     0.000000     0.948368    -0.691328
 C    -0.000000    -0.948368    -0.691328
 C     0.000000    -0.000000    -1.697139
```

Cartesian coordinates (in Å units) of the optimized geometries of isomers **1** to **11** MgC_3H^+ in their respective quartet ground electronic states obtained at the ωb97xd/6-311++G(2D,2P) level of theory.

```
    5
isomer 1 (p1a) scf done:  -314.549578
 C    -0.007373    -0.000000     1.000000
 H     1.071066    -0.000000     1.000000
 C    -1.132517    -0.000000     1.751016
 C    -1.132517     0.000000     0.248984
Mg    -3.009134     0.000000     1.000000

    5
isomer 2 (c1a) scf done:  -314.523045
 H    -0.125762     0.000000     0.034046
 C     0.021065     0.000000     1.101738
 C     0.864926     0.000000     2.127805
 C    -0.549533     0.000000     2.383125
Mg     2.515637    -0.000000     3.262359

    5
isomer 3 (c5a) scf done:  -314.512381
 C     0.074892     0.000000    -0.153255
 C     0.307357     0.000000     1.168784
Mg     2.162365    -0.000000     0.179590
 C     1.134989    -0.000000     2.149051
 H    -0.913956     0.000000    -0.599797

    5
isomer 4 (c1e) scf done:  -314.499580
Mg    -0.506926     0.000000    -0.039215
 C     0.262875    -0.000000     1.867127
```

```
 C     1.553807    -0.000000     2.135444
 C     2.741510    -0.000000     2.488684
 H     3.698279    -0.000000     2.962893

    5
isomer 5 (p1b) scf done:  -314.415900
Mg     0.245739    -0.000000     1.000000
 H     1.903220    -0.000000     1.000000
 C    -2.051758     0.000000     1.888457
 C    -2.051758    -0.000000     0.111543
 C    -2.978121     0.000000     1.000000

    7
isomer 6 (c4d) scf done:  -314.441287
 C     0.027093     0.000000     1.000000
 C     1.346034     0.000000     1.000000
 C     2.592993     0.000000     1.000000
Mg     4.843297     0.000000     1.000000
 H     6.500312     0.000000     1.000000
XX     1.346034     1.000000     1.000000
XX     2.592993     1.000000     1.000000

    6
isomer 7 (c2a) scf done:  -314.397471
Mg    -0.002014    -0.000000     1.000000
 H     1.650885    -0.000000     1.000000
 C    -2.399468     0.000000     1.000000
 C    -3.027526     0.000000     2.151090
 C    -3.027526    -0.000000    -0.151090
XX    -0.002014    -0.000000     2.000000

    8
isomer 10 (c4a) scf done:  -314.490838
Mg     0.298077     0.000000     1.000000
 C     2.220434     0.000000     1.000000
 C     3.511132     0.000000     1.000000
 C     4.757326     0.000000     1.000000
 H     5.822760     0.000000     1.000000
XX     3.511132     1.000000     1.000000
XX     2.220434     1.000000     1.000000
XX     4.757326     1.000000     1.000000

    8
isomer 11 (c4b) scf done:  -314.349061
 C    -0.310198     0.000000     1.000000
Mg     2.143069     0.000000     1.000000
 C     4.097716     0.000000     1.000000
 C     5.306100     0.000000     1.000000
 H     6.373042     0.000000     1.000000
XX     2.143069     1.000000     1.000000
XX     4.097716     1.000000     1.000000
XX     5.306100     1.000000     1.000000
```

Cartesian coordinates (in Å units) of the optimized geometries of isomers **1** to **11** MgC_3H^+ in their respective quartet ground electronic states obtained at the Uωb97xd/6-311++G(2D,2P) level of theory.

```
   5
isomer 1 scf done:  -314.468357
 C      0.068581     0.000000     1.000000
 H      1.150843     0.000000     1.000000
 C     -1.041570     0.000000     1.777958
 C     -1.041570     0.000000     0.222042
Mg     -3.346759     0.000000     1.000000

    5
isomer 2 scf done:  -314.508381
 H      0.148918     0.000000     0.009546
 C     -0.059551     0.000000     1.067960
 C      0.525682     0.000000     2.364538
 C     -0.822440     0.000000     2.149693
Mg      2.454211     0.000000     3.584798

    5
isomer 5 scf done:  -314.385517
Mg      0.000000     0.000000     0.274191
 H      0.000000     0.000000     1.926287
 C      0.683535     0.000000    -2.161602
 C     -0.683535     0.000000    -2.161602
 C      0.000000     0.000000    -3.336012

    8
isomer 6 scf done:  -314.384232
 C      0.070655     0.000000     1.000000
 C      1.335756     0.000000     1.000000
 C      2.615614     0.000000     1.000000
Mg      4.814038     0.000000     1.000000
 H      6.473666     0.000000     1.000000
XX      1.335756     1.000000     1.000000
XX      2.615614     1.000000     1.000000
XX      4.814038     1.000000     1.000000

    6
isomer 7 scf done:  -314.412048
Mg      0.135494     0.000000     1.000000
 H      1.794564     0.000000     1.000000
 C     -2.109366     0.000000     1.000000
 C     -3.313171     0.000000     1.672735
 C     -3.313171     0.000000     0.327265
XX      0.135494     0.000000     2.000000

    5
isomer 8 scf done:  -314.461203
 H      0.000000     0.000000    -0.189167
 C      0.000000     0.000000     0.893078
Mg      0.000000     0.000000     3.736769
 C     -0.944126     0.000000     1.879775
 C      0.944126     0.000000     1.879775
```

```
    8
isomer 10 scf done:  -314.528251
Mg     0.296926     0.000000     1.000000
 C     2.218245     0.000000     1.000000
 C     3.507409     0.000000     1.000000
 C     4.760995     0.000000     1.000000
 H     5.826153     0.000000     1.000000
XX     3.507409     1.000000     1.000000
XX     2.218245     1.000000     1.000000
XX     4.760995     1.000000     1.000000

    8
isomer 11 scf done:  -314.387089
 C    -0.283606     0.000000     1.000000
Mg     2.141282     0.000000     1.000000
 C     4.089907     0.000000     1.000000
 C     5.297608     0.000000     1.000000
 H     6.364540     0.000000     1.000000
XX     2.141282     1.000000     1.000000
XX     4.089907     1.000000     1.000000
XX     5.297608     1.000000     1.000000
```
